# Up-regulation of miR-9 expression predicate advanced clinicopathological features and poor prognosis in patients with hepatocellular carcinoma

**DOI:** 10.1186/s13000-014-0228-2

**Published:** 2014-12-31

**Authors:** Lizhi Cai, Xi Cai

**Affiliations:** Department of Hepatobiliary Surgery, Jingzhou First People’s Hospital/The First Affiliated Hospital of Yangtze University, No.8 Hangkong Road, Shashi district, Jingzhou, 434000 Hubei China; Academy Office, Clinical Medical College, Yangtze University, Jingzhou, China

## Abstract

**Background:**

MicroRNAs (miRNAs) are endogenous small (19–24 nt long) noncoding RNAs that regulate gene expression in a sequence specific manner. An increasing association between miRNA and cancer has been recently reported. Hepatocellular carcinoma (HCC), as the fifth most common cancer and the most common cause of death in men, has become the third leading cause of cancer-related deaths globally. In this study, we investigated the miR-9 expression in HCC to evaluate their value in prognosis of this tumor.

**Methods:**

The expression of miR-9 in matched normal and tumor tissues of HCC was evaluated using a quantitative real-time RT-PCR. A Kaplan–Meier survival curve was generated following a log-rank test.

**Results:**

It was observed that miR-9 expression was upregulated in HCC tissues compared with noncancerous liver tissues (7.26 ± 1.30 vs. 3.14 ± 1.08, P < 0.001). The up-regulation of miR-9 in HCC cancer tissues was also significantly correlated with aggressive clinicopathological features. We found that the patients with high miR-9 expression have a higher tumor staging (P = 0.0389) and are in higher risk of venous infiltration (P < 0.0001). Moreover, the results of Kaplan–Meier analyses showed that HCC patients with the high miR-9 expression tend to have shorter overall survival (P < 0.0001). The multivariate analysis clearly indicated that the high miR-9 expression in biopsy samples may be considered as an independent prognostic factor in HCC for decreased survival (4.28; 95%CI, 2.77-7.23, P < 0.001).

**Conclusion:**

Our data indicate the potential of miR-9 as a novel prognostic biomarker for HCC. Large well-designed studies with diverse populations and functional evaluations are warranted to confirm and extend our findings.

**Virtual Slides:**

The virtual slide(s) for this article can be found here: http://www.diagnosticpathology.diagnomx.eu/vs/13000_2014_228

## Background

Hepatocellular carcinoma (HCC) has become the third leading cause of cancer-related deaths globally [1]. The incidence of HCC is still increasing in the developing countries, especially in East and South-East Asia, with the highest reported primary liver cancer rates. Annually, more than 700,000 new cases are diagnosed worldwide and unfortunately more than 600,000 cases dead due to this cancer [2]. The five-year survival rate of this cancer is merely 7% [3]. The development and progression of HCC is a multistage process involving the deregulation of genes that are crucial to cellular processes, such as cell cycle control, cell growth, apoptosis and cell migration. HCC is highly lethal because of its aggressive metastasis and an advanced stage at the time of diagnosis. The occurrence of HCC is a multi-factor and multi-stage process, including both hereditary and environmental factors. Since the diagnosis at early stage of HCC offers the only hope for curative therapies, it is of utmost importance to screen high-risk patients effectively. With the increasing understanding of tumor biology of HCC, recent studies have identified more and more molecular markers with high sensitivity and specificity for diagnosis and prognosis in patients with HCC.

MicroRNA (miRNA) belongs to a class of endogenously expressed, non-coding small RNA and contains about 22 nucleotides, which exhibits a high degree conservation of structure and function in metazoa. They exist in two forms of pre-miRNAs and mature miRNAs, and only the mature miRNAs mediated by the two RNase III endonucleases Dicer and Drosha play a key biological role [4]. The mature miRNAs inhibit protein translation through binding the 3′-untranslated region (3′-UTR) of target mRNA partly, while they induce target mRNA cleavage through binding mRNA with perfect complementarity [5,6]. At present, approximately 450 miRNAs have been cloned in mammalian cells, and it is believed that up to 1,000 miRNAs genes exist [7,8]. Moreover, it is estimated that 30% genes of the human genome are regulated by miRNAs [9]. Though the biological functions of a small amount of identified miRNAs are elucidated, miRNAs have been proved to be important for cell growth, differentiation, and apoptosis [10,11]. miRNAs also regulate oncogenesis because they are both oncogenic and tumor suppressors. miRNAs regulate posttranscriptional expression of target genes involved in any biological processes including development, differentiation, cell proliferation, apoptosis, and the stress response [12]. In addition, miRNAs are involved in cancer development and progression and are differentially expressed in normal tissues and cancers.

MiR-9 is a regulator of neuronal progenitor cell fate during neurogenesis [13,14], which has recently been implicated in cancer [15]. Although most studies indicate a tumour-suppressor activity for miR-9 in cancer cells [16], conflicting data exist, and the outcome of miR-9 function appears to be tumour specific [17]. miR-9 is downregulated in breast cancer, renal cell carcinoma, and gastric cancer due to promoter methylation [18-20]. In contrast, miR-9 has been found to be upregulated in gliomas and in colorectal cancer [21,22]. Recent studies also report that miR-9 is heterogeneously expressed within a given tissue [23]. miR-9 has also recently been shown to be associated with metastasis formation in several cancer types, such as breast, colon, ovarian, cervix, liver, and gastric cancer [16,22,24-26]. Recent studies have shown that miR-9 promotes metastasis formation [22,24,26], however, in contrast, other studies have suggested that increased expression of miR-9 suppresses metastasis formation [16,25] and that miR-9 inhibits tumor growth through inhibition of NFkappa B1 [27,28]. Therefore, miR-9 might have different functions in different cancers. In this study, we investigated the miR-9 expression in HCC tissues to evaluate their value in prognosis of this tumor.

## Methods

### Samples and cases

200 pairs of samples (including 200 HCC samples and normal adjacent tissues) from HCC patients were collected from April 2004 to April 2009 at the first affiliated hospital of Yangtze University. None of the patients recruited in this study had chemotherapy or radiotherapy before the surgery. The miR-9 quantitative analysis was performed with those samples via real-time PCR. The study was approved by the first affiliated hospital of Yangtze University. Written informed consent was obtained for the acquisition and use of patient tissue samples and anonymized clinical data.

**Figure 1:**
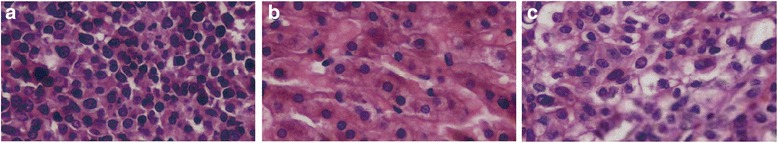
**Tumor differentiation according to the Edmondson grading system.** Poor differentiation **(a)**; Moderate **(b)**; and Well **(c)**.

The diagnosis and histological grade of each case were confirmed by two pathologists independently. The clinical stage was classified according to the Edmondson grading system. Liver function was assessed using the Child-Pugh scoring system. Tumor staging was determined according to the Union for International Cancer Control (UICC) criteria (7th Edition) and WHO classification (Pathology and Genetics of Tumors of the Digestive System). Tumor differentiation was defined as poor, moderate, and well according to the Edmondson grading system (Figure 1).

### RNA extraction

For real-time PCR analysis of miRNA, total RNA was isolated using Trizol reagent (Invitrogen) according to the manufacturer’s instructions. Briefly, plugs were punched out (1.5 mm × 1.5 mm) of a paraffin block. Samples were deparaffinized three times in 1 mL ACS grade xylene with incubation at 60°C for 10 min, followed by a wash with 100% ACS grade ethanol and air drying at room temperature. Samples were then incubated with proteinase K (Merck) at 55°C overnight, shaking every 2 h. RNA samples were resuspended in RNase-free water after the final precipitation step. RNA quality and quantity were assessed using a biophotometer (Eppendorf). The paraffin plugs were enriched for tumor tissue under microscope control using H & E-stained sections of the same sample for guidance.

The RNA concentration and purity were assessed by UV spectrophotometry (A260/A280 ratio of 1.8 - 2.0). Total RNA samples were reverse transcribed to cDNA using a TaqMan® microRNA assay miRNA-specific stem-loop primer and the TaqMan® microRNA Reverse Transcription Kit (Applied Biosystems, Foster City, CA, USA). The PCR was performed using the TaqMan® Universal PCR Master Mix and a 7500 Sequence Detection System (Applied Biosystems) according to the manufacturer’s instructions, and previously published primer sequences [29]. The cycling programme involved preliminary denaturation at 95°C for 10 min, followed by 40 cycles of denaturation at 95°C for 15 s, annealing at 60°C for 60 s and elongation at 60°C for 60 s. The U6 small nuclear (sn) RNA was amplified as an internal control using previously published primer sequences. Each sample was analysed in triplicate. Levels of miR-9 were analysed quantitatively relative to U6 snRNA by the 2^–∆∆CT^ method using the equation, relative quantity = 2^–∆∆CT^, where ∆∆CT = (CT ^miR-21^ - CT ^U6^)_cancer_- (CT ^miR-21^ - CT ^U6^) _normal adjacent tissues_, and CT is the cycle threshold for each specimen.

### Statistical analyses

To analyze baseline characteristics, the continuous variables was presented as Mean ± SD and compared between groups by the Student’s t‑tests, and the categorical data compared by Chi-square tests. Associations between miR-9 expression and over survival of the patients with HCC were estimated using adjusted relative risks and 95% confidence intervals (95% CIs) from multivariate logistic regression. Survival time was calculated from the date of HCC diagnosis to the date of death or last follow-up. Survival analysis was estimated using the Kaplan–Meier method, log-rank test, and Cox-proportional hazards regression model. The P < 0.05 was considered to indicate a statistically significant difference. The software of SPSS version13.0 for Windows (SPSS, Inc., Chicago, IL) was used for statistical analysis.

## Results

### MiR-9 expression in HCC tissues

**Figure 2:**
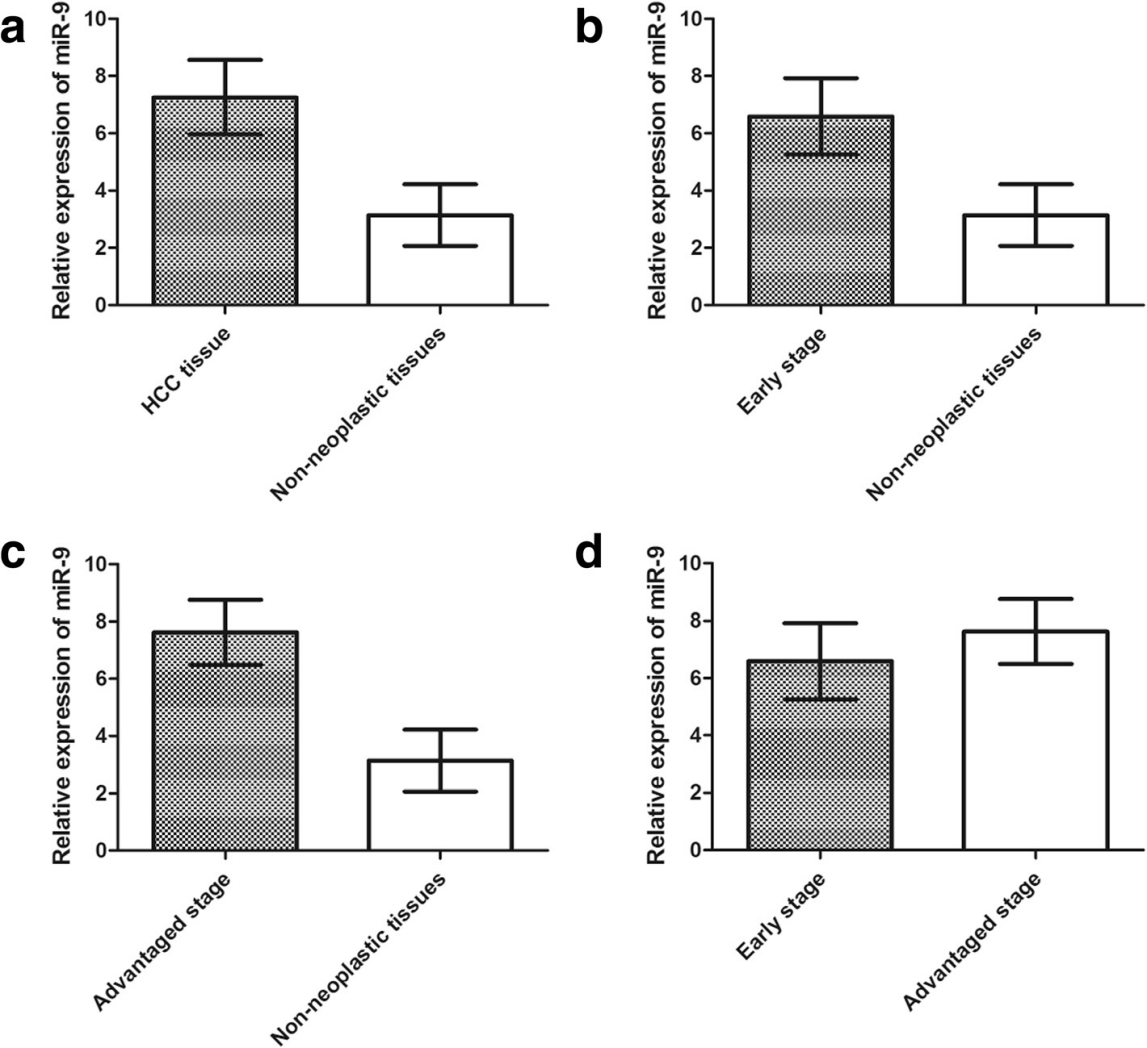
**miR-9 expression in 200 pairs of HCC and adjacent non-neoplatic liver tissues detected by quantitative real-time polymerase chain reaction (qRT-PCR) analysis.** **(a)** Comparison between all the HCC tissues and non-neoplastic tissues. **(b)** Comparison between early stage HCC tissues and non-neoplastic tissues. **(c)** Comparison between advantaged stage HCC tissues and non-neoplastic tissues. **(d)** Comparison between early and advantaged stage HCC tissues.

MiR-9 expression was detected in 200 pairs of HCC tissues and adjacent non-neoplastic liver tissues normalized to RNU6B. As shown in Figure 1, we found that the expression of miR-9 was markedly increased in HCC tissues compared with non-neoplastic liver tissues (mean ± SD: 7.26 ± 1.30 vs. 3.14 ± 1.08, P < 0.001, Figure 2a). In addition, miR-9 expression in early stage (6.59 ± 1.33) and advanced stage (7.68 ± 1.13) HCC tissues were both significantly higher than that in non-neoplastic liver tissues (3.14 ± 1.08; P < 0.0001 and <0.0001, respectively, Figure 2b and c). There was also a significant difference in miR-9 expression between early stage and advanced stage HCC tissue specimens (P < 0.001, Figure 2d).

### Expression of miR-9 and clinicopathological features

We then analyzed the association between miR-9 expression and clinicopathological parameters in HCC. HCC tissues expressing miR-9 at levels less than the median expression level (7.293) were assigned to the low expression group (mean expression value 6.22, n = 100), and those samples with expression above the median value were assigned to the high expression group (mean expression value 8.30, n = 100). The high level of miR-9 expression was significantly more common in HCC tissues with advanced pathologic grade than those with low pathologic grade (P = 0.0389, Table 1). Besides, miR-9 up-regulation group has a higher rate of venous infiltration (P < 0.0001). In this study, no significant association between miR-9 expression and tumor size was detected (P = 0.252). When the AFP status was considered, the expression of miR-9 was not different among the high and low level status (P = 0.127). No significant association was found between miR-9 expression and gender or age at diagnosis.

**Table 1:** **Correlation of miR-9 expression with clinicopathological features of HCC (n = 200)**

**Clinicopathological features**		**No. of cases**	**miR-9 expression**		***P***
			**Low**	**High**	
Age (year)	<50	78	40	38	0.662
	≥50	122	58	64	
Gender	Male	107	53	54	0.889
	Female	93	45	48	
Tumor size	<5 cm	82	36	46	0.252
	≥5 cm	118	62	56	
Tumor stage	Early stage (I-II)	71	42	29	0.0389
	Advanced stage (III-IV)	129	56	73	
Venous infiltration	Absent	131	82	49	<0.0001
	Present	69	16	53	
AFP level	≤20 ng/ml	45	27	18	0.127
	>20 ng/ml	155	71	84

**Table 2:** **Multivariate analyses of different prognostic parameters in patients with HCC by Cox regression analysis**

**Parameter**	**Risk ratio** ^**1**^	**95% CI** ^**2**^	***P*** ^**3**^
**Age**	1.60	0.40-2.65	0.667
**Gender**	1.53	0.50-2.95	0.754
**Tumor size**	2.25	0.95-4.34	0.078
**AFP**	1.38	0.58-2.64	0.430
**Venous infiltration**	2.61	1.34-5.12	0.006
**miR-9 expression**	4.28	2.77-7.23	<0.001

^1^RR; relative risk.

^2^CI; confidence interval.

^3^P-value,0.05 statistically significant and significant p-values are in bold.

### MiR-9 expression and survival in patients with HCC

**Figure 3:**
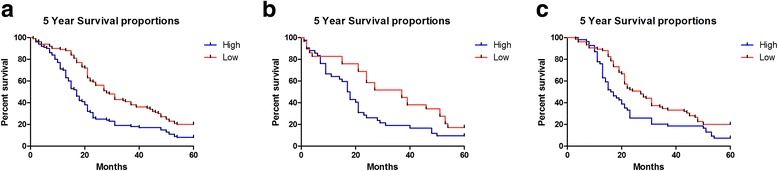
**Kaplan-Meier survival curves for HCC patients with high or low expression of miR-9. (a)** The 5-year overall survival rate of all 200 HCC patients with high or low miR-9 expression; **(b)** The 5-year overall survival rate of 71 HCC patients with early stage in high or low miR-9 expression group; **(c)** The 5-year overall survival rate of 129 HCC patients with advanced stage in high or low miR-9 expression group.

In the 5 years’ follow-up, the association between miR-9 expression and prognosis was detected using Kaplan–Meier method and log-rank test. The overall survival of HCC patients with high miR-9 expression was significantly shorter than that with low miR-9 expression (P < 0.0001, Figure 3a). When the pathological stages were considered, the higher miR-9 expression was a risk of poor prognosis in both the early stage of HCC (P = 0.0228, Figure 3b) and the advanced HCC (P = 0.0062, Figure 3c).

### Multivariate Cox proportional hazard analysis

In the Cox proportional hazard model, it was confirmed that miR-9 expression in the biopsy samples (RR 4.28; 95% CI, 2.77-7.23), tumor stage (RR 3.10; 95% CI, 1.22-4.77), and venous infiltration (RR, 2.61; 95% CI, 1.34-5.12) were predictor of poor prognosis of the patients with HCC. Our results showed that age (RR, 1.60; RR, 0.40-2.65), gender (RR, 1.53; 95% CI, 0.50-2.95), tumor size (RR, 2.25; 95% CI, 0.95-4.34), and AFP (RR, 1.38; 95% CI, 0.58-2.64) were not independent predictor of the survival of patients with HCC (Table 2).

## Discussion

Although with the development of early diagnosis and surgical techniques, the patients with hepatocellular carcinoma still results with a relatively bad prognosis. The molecular biomarkers for early diagnosis and predictor of prognosis are desperately required now. A lot of work has been conducted in an attempt to identify biomarkers with diagnostic and prognostic implications for HCC. MiRNAs have been demonstrated to be critical regulators of carcinogenesis and tumor progression in HCC. The role of miR-9 as a prognostic factor has been recently described in different tumours, such as acute lymphocytic leukemia [30], acute myeloid leukemia [31], and colon cancer [32]. Similarly in the present study, we observed that miR-9 expression was up-regulated in HCC tissues compared with noncancerous liver tissues. The up-regulation of miR-9 in HCC cancer tissues was also significantly correlated with aggressive clinicopathological features. We found that the patients with high miR-9 expression have an advanced tumor staging and are in higher risk of venous infiltration. Moreover, the results of Kaplan–Meier analyses showed that HCC patients with the high miR-9 expression tend to have shorter overall survival and progression free survival. The multivariate analysis clearly indicated that the high miR-9 expression in biopsy samples may be considered as an independent prognostic factor in HCC for decreased survival.

MiR-9 is a regulator of neuronal progenitor cell fate during neurogenesis [13,14], which has recently been implicated in cancer [15]. Although most studies indicate a tumour-suppressor activity for miR-9 in cancer cells [16], conflicting data exist, and the outcome of miR-9 function appears to be tumour specific [17]. Recent studies also report that miR-9 is heterogeneously expressed within a given tissue [23]. miR-9 is downregulated in breast cancer, renal cell carcinoma, and gastric cancer due to promoter methylation [18-20]. The transcriptional activity of miR-9 seems to be related to gene silencing by DNA methylation. Previous studies have demonstrated that the high frequency of hypermethylated CpG islands at miR-9 genes resulted in down-regulation of miR-9 in human cancer [20]. In addition, microRNA-9 is a methylation-silenced tumour suppressor that could be a potential candidate predictive marker for poor prognosis of medulloblastoma. Loss of microRNA-9 may confer a proliferative advantage to tumour cells, and it could possibly contribute to disease pathogenesis [33]. On the other hand, the down-expression of miR-9 may be introduced by hypoxic stress, resulting in alternative splicing shift to proangiogenic isoforms of VEGF165 [34]. However, in contrast, miR-9 has been found to be upregulated in various cancer tissues like HCC, gliomas, and colorectal cancer [21,22,35]. Previous study reported miR-9 may possibly promote HCC migration and invasion through regulation of KLF17 [35]. In the present study, the up-expression of the miR-9 demonstrated aggressive clinicopathological features, with the mechanism still unknown. Ma et al. have suggested a regulatory pathway of miR-9 as a metastasis-promoting miRNA [24]. They demonstrated that miR-9 directly targets CDH1, the E-cadherin- encoding mRNA, leading to down-regulation of E-cadherin and increase in cancer cell motility and invasiveness [24]. Besides induction of Epithelial-mesenchymal transition (EMT), miR-9 was found to contribute to tumor angiogenesis, another important mechanism in metastasis, through activation of beta-catenin signaling pathway induced by E-cadherin down-regulation [24]. Recently, they also showed that miR-9 can function as a metastasis-promoting miRNA even in the E-cadherin-negative breast cancer cells through downregulation of leukemia inhibitory factor receptor [36].

## Conclusion

In conclusion, our results have demonstrated that the levels of miR-9 are higher in HCC tissues than those in matched normal liver tissues and correlated with disease stage and the presence of venous infiltration. These findings enhance our understanding of the role of miR-9 in HCC progression and suggest that miR-9 may function as microtumor promoter genes in HCC. These findings suggest the potential clinical use of microRNA measurements, particularly in estimating prognosis for patients with HCC. Large well-designed studies with diverse populations and functional evaluations are warranted to confirm and extend our findings. Examining new targets and other biological experiments will clarify the functions and roles of microRNAs in HCC.
